# China’s terrestrial ecosystem carbon balance during the 20th century: an analysis with a process-based biogeochemistry model

**DOI:** 10.1186/s13021-022-00215-9

**Published:** 2022-10-08

**Authors:** Yanyu Lu, Yao Huang, Qianlai Zhuang, Wei Sun, Shutao Chen, Jun Lu

**Affiliations:** 1 Anhui Province Key Laboratory of Atmospheric Science and Satellite Remote Sensing, Anhui Institute of Meteorological Sciences, Hefei, China; 2 Shouxian National Climatology Observatory, Huaihe River Basin Typical Farm Eco- meteorological Experiment Field of CMA, Shouxian, China; 3 College of Engineering & Natural Sciences, The University of Tulsa, Tulsa, OK USA; 4 State Key Laboratory of Vegetation and Environmental Change, Institute of Botany, Chinese Academy of Sciences, Beijing, China; 5 Department of Earth, Atmospheric, and Planetary Sciences, Purdue University, West Lafayette, IN USA; 6 School of Applied Meteorology, Nanjing University of Information Science and Technology, Nanjing, China

**Keywords:** China, Terrestrial ecosystem Model, Carbon cycle, Climate change, Land use, Hu Huanyong line

## Abstract

**Background:**

China’s terrestrial ecosystems play a pronounced role in the global carbon cycle. Here we combine spatially-explicit information on vegetation, soil, topography, climate and land use change with a process-based biogeochemistry model to quantify the responses of terrestrial carbon cycle in China during the 20th century.

**Results:**

At a century scale, China’s terrestrial ecosystems have acted as a carbon sink averaging at 96 Tg C yr^− 1^, with large inter-annual and decadal variabilities. The regional sink has been enhanced due to the rising temperature and CO_2_ concentration, with a slight increase trend in carbon sink strength along with the enhanced net primary production in the century. The areas characterized by C source are simulated to extend in the west and north of the Hu Huanyong line, while the eastern and southern regions increase their area and intensity of C sink, particularly in the late 20th century. Forest ecosystems dominate the C sink in China and are responsible for about 64% of the total sink. On the century scale, the increase in carbon sinks in China’s terrestrial ecosystems is mainly contributed by rising CO_2_. Afforestation and reforestation promote an increase in terrestrial carbon uptake in China from 1950s. Although climate change has generally contributed to the increase of carbon sinks in terrestrial ecosystems in China, the positive effect of climate change has been diminishing in the last decades of the 20th century.

**Conclusion:**

This study focuses on the impacts of climate, CO_2_ and land use change on the carbon cycle, and presents the potential trends of terrestrial ecosystem carbon balance in China at a century scale. While a slight increase in carbon sink strength benefits from the enhanced vegetation carbon uptake in China’s terrestrial ecosystems during the 20th century, the increase trend may diminish or even change to a decrease trend under future climate change.

**Supplementary Information:**

The online version contains supplementary material available at 10.1186/s13021-022-00215-9.

## Background

Terrestrial ecosystems play an important role in the global carbon (C) cycle and have been found to act as net carbon sinks, offsetting 10–60% of the CO_2_ emitted through human activities over last decades [1, 2]. As a country with vast territory area and the world’s largest population, China significantly contributes to the global carbon balance in terms of both carbon emission Boden et al., [3] and carbon uptake [4]. Since 2006, China has become the largest CO_2_ emitter, accounting for 27% of global emissions [5]. Whether China can effectively contribute to the mitigation of global warming depends on the magnitude of net C balance [6], highlighting the need to adequately quantify the carbon balance of terrestrial ecosystems C cycle in China.

China’s terrestrial ecosystems have been recognized as a substantial carbon sink in recent decades [4, 6–8]. However, there was a remarkable uncertainty on whether the terrestrial ecosystems in China are sinks or sources for atmospheric CO_2_ because they depend heavily on changing climate and land use as well as atmospheric CO_2_. Over the past century, the average air temperature in China has increased by 0.5 to 0.8 °C [9], which is higher than the global average [10]. The timing and intensity of climate change in China tend to be above the global average level [11]. The changes and feedbacks of East Asia summer monsoon systems [12] may even accelerate the climatic changes in this region. Furthermore, China has also been experiencing a dramatic land-use change that disturbs terrestrial carbon storage and fluxes and consequently has a particularly long-term impact on the carbon cycle at regional scales [7, 13]. Chinese terrestrial ecosystems likely play a pronounced role in the global carbon dynamics in a changing climate [4, 14] and human society, which have drawn much attention from both the public and scientific community. To date, considerable efforts and progress have been made to improve our knowledge about China’s carbon budget and its impact factors. However, due to the complex response and feedback of terrestrial carbon cycle to climate change and the anthropogenic disturbance, large uncertainties still exist in the carbon budget [6]. The mechanisms and factors that govern the terrestrial carbon cycle are not yet fully accounted for in quantify the regional carbon budget [15]. In particular, the relationships between individual controlling factor and the spatial and temporal variabilities of carbon balance in China are still poorly quantified under the condition of concurrent changes and complex interactions of the major factors. Furthermore, to date, most studies have focused on the last several decades of the 20th century because the dramatic changes have been recognized to occur in the corresponding periods. Meanwhile, relatively little attention has been paid to the carbon budget of China’s terrestrial ecosystems at a century scale. The role of ecosystems in carbon cycle during the early 20th century should not be ignored even if the relatively weak variabilities, because quantifying long-term terrestrial carbon dynamics will further help us to comprehensively understand the natural and anthropogenic contribution to global carbon cycle and climate change. Moreover, estimating the magnitudes of carbon fluxes at a century scale is critical for evaluating the ecosystem goods and services and cumulative contribution of regional ecosystems to carbon sequestration [16–18] and providing a scientific basis for international climate change negotiations [19]. The research is therefore of great importance to public as well as to policy makers.

Here we apply an extant process-based biogeochemistry model, the Terrestrial Ecosystem Model (TEM) [20, 21] to quantify the carbon budget for China’s terrestrial ecosystems during 1900–2000. Using the spatially explicit historical dataset, we examine how climate, atmospheric CO_2_ concentrations and land-use changes have collectively affected terrestrial C dynamics in China. The study also strives to identify the key controls to spatial and temporal patterns of C dynamics in this region.

## Methods

### The terrestrial ecosystem Model

The TEM is a process-based, global-scale ecosystem model that uses spatially referenced information on climate, elevation, soils, and vegetation to make monthly estimates of C fluxes and pool sizes of the terrestrial biosphere. In TEM, for each time step, net ecosystem productivity (NEP) is calculated as the difference between net primary productivity (NPP) and heterotrophic respiration (RH). NPP is calculated as the difference between gross primary production (GPP) and plant autotrophic respiration (RA). The structure, algorithm, parameterization, calibration and performance of TEM have been well documented [20–24].

Although many of the parameters in the model are defined from published information [20, 22], some are determined by calibrating the model to fluxes and pool sizes of intensively studied field sites. For this application of TEM in China’s terrestrial ecosystems, a set of parameters have been developed based on field observation data collected from different types of ecosystem at different regions [20, 21]. After calibration, the TEM was validated to be able to well reproduce the observed ecosystem C dynamics at site level in this region [25–27], which provides a sound basis for the application of TEM in this region.

### Spatially explicit data organization for simulation

To run TEM in China for the 20th century, we organize the data of atmosphere, vegetation, ratio of croplands, soil texture, and elevation at 0.5° latitude x 0.5° longitude resolution from 1900 to 2000. Specifically, the vegetation data over China are derived from the International Geosphere-Biosphere Program (IGBP) Data and Information System (DIS) DISCover Database [28] and re-classified into the TEM vegetation classification scheme [23]. The soil texture data are based on the Food and Agriculture Organization/Civil Service Reform Committee (FAO/CSRC) digitization of the FAO-UNESCO soil map. For elevation, the 1 km x 1 km elevation data derived from the Shuttle Radar Topography Mission (SRTM) [29] are adopted and re-sampled to match the resolution of other input data.

The driving climate data sets include the monthly air temperature, precipitation, and cloudiness. The historical climate data sets from 1900 to 2000 are based on the data from the CRU [30]. The annual atmospheric CO_2_ concentrations data from 1900 to 2000 are based on observation and our previous studies [20, 21]. The spatially explicit dataset concerning dynamic cropland change from 1900 to 2000 were derived from the historical reconstruction dataset by Yu et al. [31]. For other land types, we first subtract the area of each grid from the area of cropland, and then allocate the remaining area to other land types by referring to the ratio in HYDE dataset during 1900 ~ 2000 [32].

### Regional simulation

For each grid cell, we first run TEM to equilibrium for an undisturbed ecosystem using the long-term averaged monthly climate and CO_2_ concentrations from 1900 to 2000. We then spin up the model for 150 years with the climate from 1900 to 1949 to account for the influence of inter-annual climate variability on the initial conditions of the undisturbed ecosystem. We finally run the model with transient input datasets from 1900 to 2000 to quantify carbon dynamics in China’s terrestrial ecosystems.

To better understand the contributions of different factors, we used the factorial simulation approach as described by Piao et al. 2009 and Tian et al. 2011 [7, 15]. In our study, four simulation experiments were conducted to examine the effects of climate, atmospheric CO_2_ concentration, and land use change (Table 1).

**Table 1:** Description of simulation used in this study

Simulation	CO_2_ Concentration	Climate	Land Use Change
S1	1900–2000	1900 ~ 2000	1900 ~ 2000
S2	1900	1900 ~ 2000	1900 ~ 2000
S3	1900 –2000	1900 ~ 1919 average	1900 ~ 2000
S4	1900 –2000	1900 ~ 2000	1900

## Results

### Environmental changes in China during the 20th century

During the 20th century, the climatic factors controlling terrestrial carbon cycle changes substantially (Additional file 1: Fig. S1). The decadal average of air temperature was observed to increase by about 1 °C from 1900s to 1990s at the national scale, which is higher than the corresponding global average level (about 0.60 °C). This rising trend is more significant in the last three decades of the 20th century and the change rate arrived at 0.30 °C per decade. The mean precipitation over China show great inter-annual variability, while the change trend is not significant. There is a slight downward trend in cloudiness in China during the 20th century, especially in the last three decades, which implies an increase in solar radiation.

Soil thermal and moisture conditions are the two most important factors affecting ecosystem carbon balance. It has been shown in previous studies that TEM models is able to well estimate soil thermal and moisture conditions in China. Driven by the increased air temperature, soil thermal dynamics simulated by TEM also exhibit an increasing trend during the 20th century. In particular, more significant increase trend occurs in the soil temperature at the top 20 cm depth with the rate of 0.23 °C per decade for the period of 1970 to 2000 (R^2^ = 0.50; p < 0.01; n = 31). At the national scale, annual averages of volumetric soil moisture are simulated to fluctuate between 46 and 49%, mainly due to the variation of precipitation. A drying trend, however, occurs in soil moisture since the middle of the 1980s even though the decadal average of precipitation remains at almost the same level during the corresponding period.

**Fig. 1:**
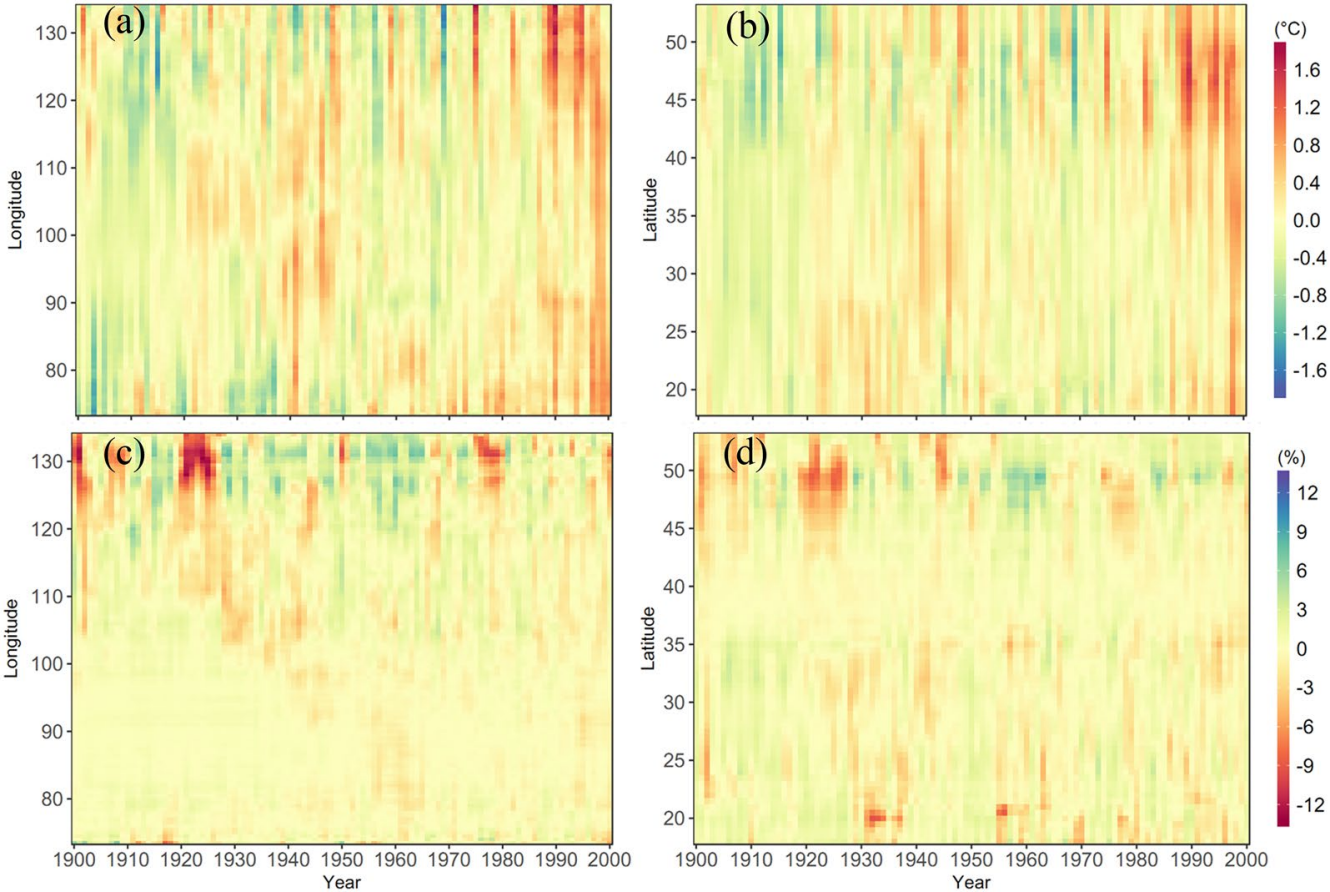
Inter-annual variation in anomalies of annual soil temperature at top 20 cm soil depth (**a**, **b**) and moisture (**c**, **d**) at different longitudes (**a**, **c**) and latitudes (**b**, **d**) from 1900 to 2000 in China

The changes in soil thermal and hydrological condition are not uniform across China. Figure 1 depicts the inter-annual variation in the simulated soil temperature and moisture at different longitudes and latitudes. For soil thermal dynamics, TEM simulation indicates that soil temperature at nearly all longitudes and latitudes has significantly increased during the 20th century, in particular for its last three decades (Fig. 1a, b). However, different change rates are found across China. Specifically, in comparison to the early last century, soil temperature during the last decade has increased by above 1.60 °C in the north and east belts, which is remarkably higher than the magnitude of increase (below 1.00 °C) at the central and southern China. These spatiotemporal patterns of soil temperature changes correspond closely with the relatively large warming trend in air temperature in the northeast China [9]. For soil hydrological dynamics, the changes in soil moisture have no significant trend but generally exhibit a decadal dry-wet cycle at different longitude and latitude strips (Fig. 1c, d). A larger inter-annual variability in soil moisture has been simulated in the east and high-latitude regions. It is noteworthy that there is persistent and extensive drought, as indicated by the negative anomalies of soil moisture, from 1922 to 1932 in the northern and eastern China, which is also recorded as one of the severest drought events in history [33].

### Terrestrial ecosystem carbon dynamics during the 20th century

**Fig. 2:**
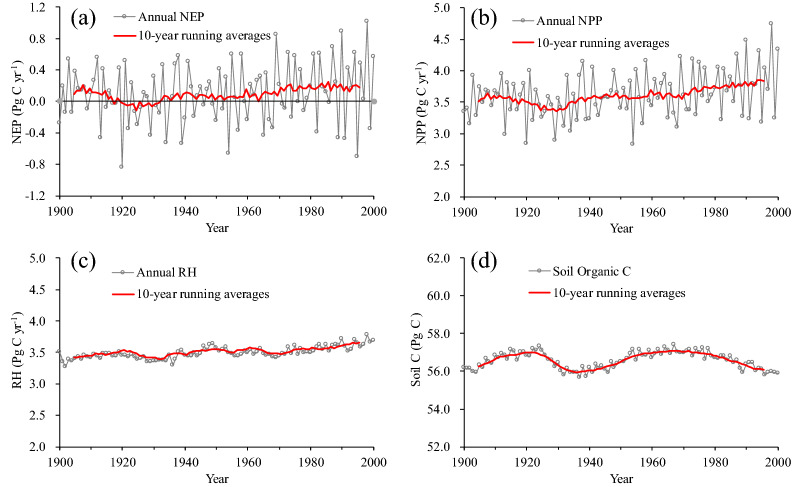
Inter-annual variability for **a** net ecosystem production (NEP), **b** net primary production (NPP), **c** heterotrophic respiration (RH) and **d** soil carbon pool from 1900 to 2000 in China

Our simulations indicate that China’s terrestrial ecosystem acted as a C sink of 96 Tg C yr^− 1^ during the 20th century (Fig. 2a). This sink is the difference between NPP of 3.61 Pg C yr^− 1^ (Fig. 2b) and heterotrophic respiration (RH) of 3.51 Pg C yr^− 1^ (Fig. 2c). The C dynamics in China however exhibit a significant inter-annual variability. The terrestrial ecosystem has been estimated as a C source in 43 years during 1900–2000. Comparing with RH, the changes in NPP fluctuate more intensively and dominate the sink-source oscillation in NEP.

Due to rising temperature, longer growing season, and the fertilization effects of increase atmospheric CO_2_ concentrations, the NPP over China increases at the rate of 38 Tg C per decade during the last century (Fig. 2b). A significant rising trend in the changes of RH is also found but at a relatively low rate (23 Tg C per decade). Consequently, as the difference of NPP and RH, the total NEP increased from 92 Tg C yr^− 1^ in the 1900s to 210 Tg C yr^− 1^ in the 1990s. However, it should be noted that since the 1970s the change rate of RH apparently accelerates in comparison to the changes in NPP. The aggravating carbon loss by RH partially offsets the increase in plant C uptake, even induces a slight decrease in C sink over China in the end of the 20th century. This suggests that the effects of rising temperature or the atmospheric CO_2_ concentrations on plant C uptake can’t keep up with ecosystem C loss in this region under the condition of intensive climate change [26, 34].

Comparing with the C fluxes, the C pool sizes in China are relatively stable, and did not show significant inter-annual variation. The relative change proportions are less than 15% through the 20th century (Fig. 2d). TEM estimates that soil C pool size average at 56.57 Pg C over the last century and 56.11 Pg C in the 1990s. A decrease trend presents in the variation of soil C pool sizes in the late 20th century, as a result of the increase in RH during the corresponding period.

### Spatiotemporal patterns of terrestrial ecosystem carbon cycle

**Fig. 3:**
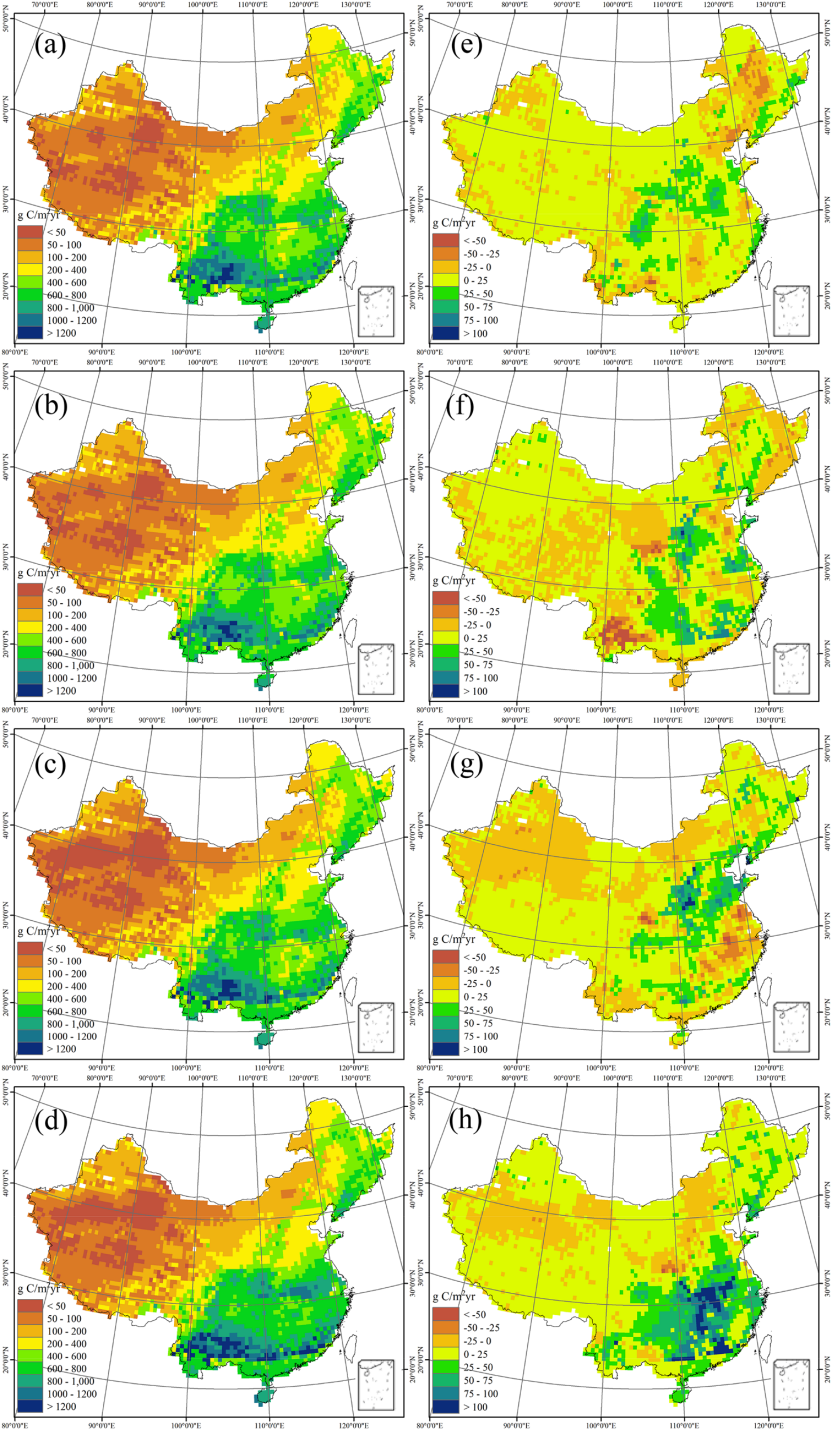
Spatial patterns of annual net primary production (NPP, **a**–**d**) and net ecosystem production (NEP, **e**–**h**) during the 1900s (**a**, **e**), 1930s (**b**, **f**), 1960s (**c**, **g**), and 1990s (**d**, **h**) in China

There is a great spatial variability in carbon dynamics in China’s terrestrial ecosystems, and the spatial pattern has also changed greatly during the 20th century (Fig. 3a ~ d). NPP is simulated to vary from less than 50 g C m^− 2^ yr^− 1^ to over 1200 g C m^− 2^ yr^− 1^ across China. The south and east regions have higher NPP and the largest value is estimated in the tropical regions around the Tropic of Cancer, while the Northwest China and Tibet Plateau are dominated by the lowest plant productivity. Interestingly, the dividing line of 400 g C m^− 2^ yr^− 1^ of NPP almost coincide with the Hu Huanyong line, a geo-demographic demarcation line which was proposed to illustrate the enormous difference in population density between the two sides of the line [35]. Further, the dividing line of NPP is simulated to have not fundamentally changed during the 20th century, and the Hu Huanyong line also remains in place [35]. The spatial congruence between net primary productivity and human population is also found in other regions [36]. According to the phases of human development that influence population distribution in relation to NPP [37], the strong link between population and productivity firstly manifests the reliance of humans on local agricultural production, and the people then still prefer to settle in regions of relatively high NPP due to population agglomeration effect even though the local environmental constraints have been weakened by industrialization and urbanization.

During the 20th century, a substantial interdecadal variation has been found in the spatial pattern of NPP. The increase in NPP is simulated to occur in the east China, while the west regions are dominated by declining NPP, especially for the northwest. The spatial distribution of NEP indicates that most of China’s terrestrial ecosystems act as a C sink (Fig. 3e–h). Specifically, the largest C sink with NEP over 100 g C m^− 2^ year^− 1^ is located in the forest ecosystems of the southern China and the croplands in eastern China, while net C loss has been found in some arid regions in the northwest. Comparing with NPP, the interdecadal change in spatial pattern of NEP exhibits a relatively large variability. The role of terrestrial ecosystems acting as a C sink or a source can be found to shift frequently in most areas during the 20th century. Generally, the areas characterized by C source are simulated to extend in the west and north of the Hu Huanyong line, since the water scarcity in these regions constrains the increase in vegetation productivity and C uptake [38, 39]. Meanwhile, the eastern and southern regions increase their area and intensity of C sink, particularly in the late 20th century.

### Contribution of different ecosystems

**Fig. 4:**
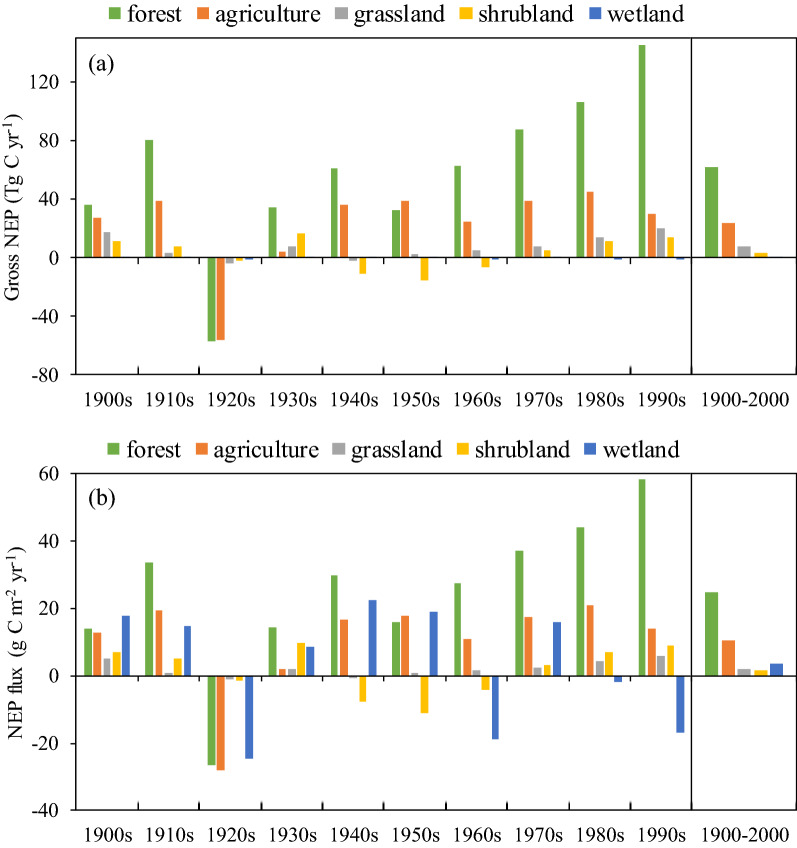
Net ecosystem production (NEP) of major ecosystem types in China during different decades. **a** gross NEP, **b** NEP on per area basis

Over the 20th century, forest ecosystems dominate the C sink in China and are responsible for about 64% of the total sink (Fig. 4a). Croplands contribute the second largest C sink of 24 Tg C m^− 2^ yr^− 1^. Grasslands and shrublands account for 8% and 3% of the total NEP, respectively. The rest of sink, less than 0.10%, is contributed by the wetlands. On a per unit area basis, forest ecosystems also have the largest C sink strengths, averaged at 25 g C m^− 2^ yr^− 1^ during the last century (Fig. 4b). Although croplands are the second largest sink strengths, the mean NEP of croplands amount to less than half of the forest. Wetlands are characterized by higher C sink strengths than grasslands and shrublands, but their small area substantially limits their gross contribution.

Driven by climatic and land use changes, different interdecadal change patterns are found for different ecosystems. Specifically, fluctuations in decadal average NEP of forest ecosystems are simulated to dominate in the early 20th century, and then a significant increase trend is found in the second half of the last century. The total NEP of forests has a relatively large increase trend, mainly due to the large-scale reforestation and afforestation since the 1970s. The change of NEP in croplands almost synchronize with forest except the remarkable decrease in the 1960 and 1990 s resulting from the high sensitivity of agriculture production to climate change and the emergence of extensively extreme events in cropland areas during the corresponding periods [40–42]. The national drought in 1960s and severe floods in 1990s have had seriously impact on agricultural production. Forest ecosystems are relatively more resistant to droughts and floods than agriculture, and these disasters occur mainly in main cropland such as central and eastern China, so the carbon uptake of forest ecosystems is less affected on a national scale. Land-use changes also affect the contribution of croplands, and the decrease in cropland area in the late 20th century diminished the increase trend of its NEP in comparison to the C uptake on per area basis. The variation of NEP in grasslands and shrublands is basically in line with that of forest ecosystems, except the role of shrublands acting as a C source during the middle of the 20th century. The flux of wetlands has the largest inter-decadal variability among these ecosystems and does not show a significant change trend. On decadal average, most ecosystems act as a C sink during the 20th century except for the 1920s. Net C loss is simulated to occur in all ecosystems in the 1920s, as a result of the persistent and extensive drought event as highlighted in “Environmental changes in China during the 20th century” section.

## Discussion

### Impact of major driving factors on carbon cycle

Spearman rank correlation analysis was used to demonstrate the response of the carbon cycle to spatial and temporal changes in the main influencing factors (Additional file 1: Table S1; Fig. S2). Among these factors, the spatial patterns of carbon fluxes are most sensitive to precipitation. The spatial patterns of NPP and NEP (Fig. 3) can be mainly explained by the distribution of precipitation. NEP dynamics in the arid and semiarid northern regions show a more significant correlation with precipitation (Additional file 1: Fig. S2), since the plant growth is relatively more water-limited in these areas [51]. The high correlation between precipitation and C dynamics is consistent with the observations in the field and satellite products [44–46], suggesting that the water availability is highly limiting the vegetation growth and soil microbe activity in China’s terrestrial ecosystems. Air temperature also reasonably influences the spatial variation of NPP and RH, while NEP has a much weaker correlation with temperature (Additional file 1: Table S1; Fig. S2). This may be attributed to the parallel positive response of NPP and RH to temperature [15]. In addition to thermal and water conditions, cloudiness is another important factor to affect terrestrial C dynamics by limiting photosynthesis [47]. Cloudiness exerts a significant control over the spatial variability in C fluxes by affecting light availability for plant growth in China (Additional file 1: Table S1). Soil properties also play an important role in terrestrial C cycle. As an example, soil texture can influence the soil thermal and moisture regimes [48], thereby impacting C storage and fluxes [49]. The negative response of C fluxes to the fraction of clay implies that loose soil texture may favor the vegetation C uptake and soil microbial activities in China. According to the partial correlation, the topography is another factor controlling the spatial pattern of C fluxes. The impact of elevation on ecological community and climate may account for the response of C fluxes to altitude changes. Together with environmental factors, human activity can also considerably influence the terrestrial C cycle through altering the land ecosystems. The shift from natural ecosystems to croplands tends to reduce the local NPP but has no significant impact on RH. Consequently, the variation in C sink strength per grid cell shows a negative response to the change of cropland area.

**Fig. 5:**
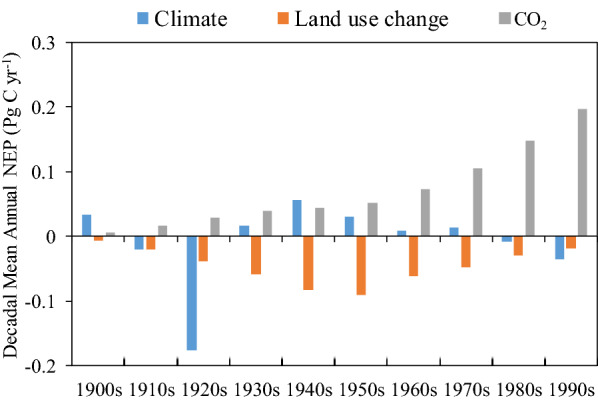
Changes in NEP from different factors changes in China from 1900 to 2000. Factors include atmospheric CO_2_ (S1-S2), climate (S1-S2), and land use change (S1–S3).

Since there may be a non-linear response relationship between the carbon cycle and the factors, we further adopted factorial simulations to investigate the contribution of climate, CO_2_ and land use change to the carbon balance. On the century scale, the increase in carbon sinks in China’s terrestrial ecosystems is mainly contributed by rising CO_2_, but 62% of its contribution is offset by the adverse effects of land use change (Fig. 5). Climate warming contributes about 10% of the increase in carbon sinks. In terms of interdecadal variation, the contribution of CO_2_ fertilization to carbon sinks continues to increase. The negative effect of land use change continues to increase in the first half of the century. Due to large-scale afforestation and reforestation, the adverse effects of land use change in the first half of the century are gradually decreasing, promoting an increase in terrestrial carbon uptake in China from 1950s [7]. Compared to CO_2_ and land use, he interdecadal variability of climate impacts is more complex. Although climate change has generally contributed to the increase of carbon sinks in terrestrial ecosystems in China, it is noteworthy that the positive effect of climate change has been diminishing since the 1940s. In particular, the impact of climate change on carbon sinks in the last two decades has shifted to negative effect. Continued warming of the climate leads to increased evapotranspiration, which in turn dries out the soil (Fig. 1, Additional file 1: Fig. S1). Declining soil moisture reduces ecosystem water availability and increases water stress, which may account for the negative effects of climate change from 1980s.

The carbon cycle of terrestrial ecosystems is influenced by multiple factor, such as climate, atmospheric CO_2_ concentration, ozone pollution, nitrogen deposition, nitrogen fertilizer application, and land use change. In particular, the application of chemical fertilizer nitrogen to agricultural fields during recent decades may have had a significant impact on terrestrial ecosystem carbon balance in China. Ideally, in order to obtain more accurate results, the impacts of these factors should be fully considered during the simulation. However, limited by factors such as data availability, we focus on the effects of climate, CO_2_ concentration, and land use change in this study. In other words, our simulations should be treated as “potential” sink or source activities, rather than an “accurate” quantification.

### Comparison with other studies

Carbon budgets of China’s terrestrial ecosystems have been quantified at different time scales by using various methods in previous studies. A comparison of our results to other studies was conducted (Additional file 1: Table S2). At the national scale, our estimates of terrestrial C uptake agreed well with other estimates. For example, Piao et al. estimated that China’s terrestrial ecosystems sequestered carbon at a rate of 186 ~ 261 Tg C yr^− 1^ from 1980 to 2002 [4]. Our simulated carbon sink of 211 Tg C yr^− 1^ is within this range. During the period of 1961–2000, TEM estimates a slightly lower C sink strength than the estimate reported by Tian et al. [7], mainly owing to not including the increase in N fertilizer application in our simulation and thereby underestimating CO_2_ sequestration in croplands. The average of NPP simulated by TEM at the century scale is 3.62 Pg C yr^− 1^ over China, which is close to a recent literature-based result [6]. For the last two decades of the 20th century, NPP is estimated to vary from 2.94 to 3.96 Pg C yr^− 1^ by different studies [4, 6], and our result is consistent with these estimates. TEM estimate of RH is slightly higher than other estimates. TEM simulated soil organic carbon storage is considerably lower than 80 Pg C that is commonly estimated in China’s terrestrial ecosystems. The underestimate of soil carbon might be partially due to parameterization with limited observational data, which has also been shown in the previous site-level verification [21].

For the contribution of different ecosystems, our modeled results are fundamentally in line with the existing estimates. Specifically, during 1981–2000, the croplands in China are simulated in our study to sequester CO_2_ at a rate of 37 Tg C yr^− 1^, which is comparable to 39 Tg C yr^− 1^ reported by Huang & Sun [52]. TEM-simulated C sink of 17 Tg C yr^− 1^ for grassland is within the range of other estimates [7]. On a per unit area basis, our simulated NEP averages at 51 g C m^− 2^ yr^− 1^ for China’s forest ecosystems, which is slightly lower yet comparable to the result based on inventory method [4].

For the variability of carbon dynamics, most studies have documented that NPP in China’s terrestrial ecosystems has significantly increased over the late 20th century. Our simulation also shows a similar trend and magnitude of change in NPP. However, divergent change trends in NEP are estimated among different studies. Similar to some previous process-based model studies [7], TEM-simulated rate of carbon accumulation in China’s terrestrial ecosystems firstly accelerates and then slows down or even slightly decreases at the end of the last century. The discrepancy for the variation in C sink strength is mainly due to the difference in the estimate of soil carbon dynamics and storages, which will consequently incur a large uncertainty in the current regional carbon budget and future projection for terrestrial ecosystems. This suggests that the research priority should be directed to investigating the key controls on and processes of soil carbon dynamics and pool sizes as well as more detailed soil thermal and hydrological dynamics under a changing climate. In addition, the interaction among human activities, climate change, and ecosystems is with an even larger uncertainty. Some important factors concerning human activities such as the land management, fertilizer application, were not addressed in our simulations, which may introduce biases in our current estimates. To improve our analysis of regional carbon dynamics, further efforts should be made to quantify how human appropriate and alter the land ecosystems and its impacts on carbon cycle in China.

## Conclusion

This study represents a new effort to explicitly examine the regional C dynamics in response to climate change and human activity in China during the 20th century. We find that China’s terrestrial ecosystems acted as a carbon sink of 96 Tg C yr^− 1^ in the last century with a large inter-annual and spatial variability. Our analysis further suggests that the historical changes in regional carbon cycle show complex responses to changes in climate, CO_2_ concentration, and land use. Driven by these factors, the regional C cycle has been accelerated, leading to an increase for both vegetation productivity and soil decomposition. Ecosystems’ C sink or source is found to be highly sensitive to the hydrological conditions. The extensive droughts have caused the net carbon losses from almost all types of ecosystems during the 1920s. Among the major ecosystem types, forest contributes most to the C sink strength in China. During the 20th century, the increase in carbon sinks in China’s terrestrial ecosystems is mainly contributed by rising CO_2_. Large-scale afforestation and reforestation promote increase in terrestrial carbon uptake in China from 1950s. Although climate change has generally contributed to the increase of carbon sinks in terrestrial ecosystems in China, the positive effect of climate change has been diminishing in the last decades of the 20th century.

## Supplementary Information

**Additional file 1: Fig. S1**. Inter-annual variability for air temperature (a), precipitation (b), soil temperature at 20 cm depth (c), and soil moisture (d) from 1900 to 2000 in China. The percentage water-filled pore space (WFPS) is used as an expression of soil moisture.** Fig. S2**. Spatial patterns of partial correlation coefficients between net ecosystem production (NEP) and (a) air temperature, (b) precipitation, (c) CO_2_ concentration, and (d) ratio of cropland area from 1900 to 2000 in China. Black cross marks that the correlation does not pass the significant test with a 95% confidence.** Table S1**. Spatial correlations between carbon fluxes (NEP, NPP, and RH) and driving variables across China.** Table S2**. Carbon fluxes (NEP, NPP, and RH) from China’s terrestrial ecosystems by different studies.

## Data Availability

The datasets during and/or analysed during the current study available from the corresponding author on reasonable request.
