# Supplementary material for: China’s terrestrial ecosystem carbon balance during the 20th century: an analysis with a process-based biogeochemistry model
Source: Carbon Balance Manag. 2022 Oct 8;17:16. doi: 10.1186/s13021-022-00215-9 (PMC9548143; doi:10.1186/s13021-022-00215-9)
Supplement: Supplementary file 1 — Additional file 1: Fig. S1. Inter-annual variability for air temperature (a), precipitation (b), soil temperature at 20 cm depth (c), and soil moisture (d) from 1900 to 2000 in China. The percentage water-filled pore space (WFPS) is used as an expression of soil moisture. Fig. S2. Spatial patterns of partial correlation coefficients between net ecosystem production (NEP) and (a) air temperature, (b) precipitation, (c) CO2 concentration, and (d) ratio of cropland area from 1900 to 2000 in China. Black cross marks that the correlation does not pass the significant test with a 95% confidence. Table S1. Spatial correlations between carbon fluxes (NEP, NPP, and RH) and driving variables across China. Table S2. Carbon fluxes (NEP, NPP, and RH) from China’s terrestrial ecosystems by different studies. [file 13021_2022_215_MOESM1_ESM.docx]

Supplementary Figure 1. Inter-annual variability for air temperature (a), precipitation (b), soil temperature at 20 cm depth (c), and soil moisture (d) from 1900 to 2000 in China. The percentage water-filled pore space (WFPS) is used as an expression of soil moisture.

(c)

(d)

(b)

(a)

Supplementary Figure 2. Spatial patterns of partial correlation coefficients between net ecosystem production (NEP) and (a) air temperature, (b) precipitation, (c) CO_2_ concentration, and (d) ratio of cropland area from 1900 to 2000 in China. Black cross marks that the correlation does not pass the significant test with a 95% confidence.


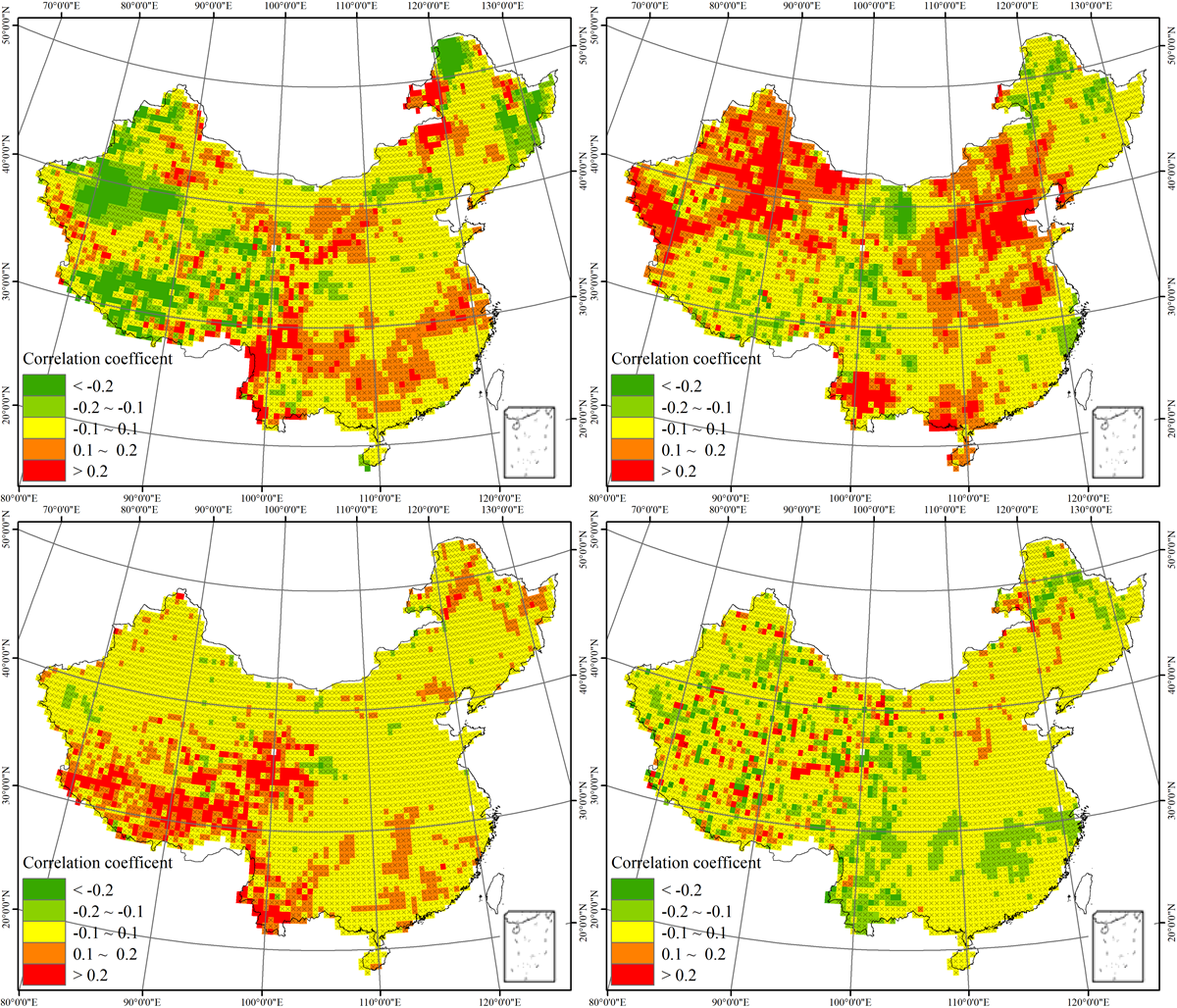


(d)

(c)

(b)

(a)

Supplementary Table 1. Spatial correlations between carbon fluxes (NEP, NPP, and RH) and driving variables across China.

|  | Air Temperature | Precipitation | Cloudiness | Soil texture | Elevation | Ratio of cropland area |
| --- | --- | --- | --- | --- | --- | --- |
| NEP | 0.06^**^ | 0.42^**^ | -0.06^**^ | -0.09^**^ | -0.17^**^ | -0.06^**^ |
| NPP | 0.28^**^ | 0.28^**^ | -0.14^**^ | -0.16^**^ | -0.09^**^ | -0.06^**^ |
| RH | 0.29^**^ | 0.26^**^ | -0.13^**^ | -0.16^**^ | -0.08^**^ | 0.02 |

** P < 0.01, *P < 0.05

The values for carbon fluxes and driving variables at each grid cell are annual averages for the 1990s except for soil texture and elevation. Soil texture is expressed as the proportion of clay.

Supplementary Table 2. Carbon fluxes (NEP, NPP, and RH) from China’s terrestrial ecosystems by different studies.

|  | Period | Fluxes  (Tg C yr^-1^) | Source |
| --- | --- | --- | --- |
| NEP | 1961~2000 | 172 | This study |
|  | 1961~2005 | 210 | Tian et al. 2011a |
|  | 1981~2000 | 211 | This study |
|  | 1980~2002 | 186~261 | Piao et al. 2009a |
|  | 1981-2000 | 100 | Ji et al. 2008 |
|  | 1981-2000 | 70 | Cao et al. 2003 |
|  | 2000s | 191 | Zhu et al. 2014 |
| NPP | 1901~2000 | 3615 | This study |
|  | 1901-2005 | 3350 | Shao et al. 2016 |
|  | 1981~2000 | 3826 | This study |
|  | 1980~2004 | 3530~3960 | Gu et al. 2017 |
|  | 1981~2000 | 2940 | Ji et al. 2008 |
| RH | 1981~2000 | 3614 | This study |
|  | 1981~2000 | 2840 | Ji et al. 2008 |
|  | 1981~2000 | 3020 | Cao et al. 2003 |

Reference:

Cao, M., Prince, S.D., Li, K., Tao, B., Small, J., Shao, X., 2003. Response of terrestrial carbon uptake to climate interannual variability in China. Global Change Biology 9, 536–546. doi:10.1046/j.1365-2486.2003.00617.x

Gu, F., Zhang, Y., Huang, M., Tao, B., Liu, Z., Hao, M., Guo, R., 2017. Climate-driven uncertainties in modeling terrestrial ecosystem net primary productivity in China. Agric. For. Meteorol. 246, 123–132. doi:10.1016/j.agrformet.2017.06.011

Ji, J., Huang, M., Li, K., 2008. Prediction of carbon exchanges between China terrestrial ecosystem and atmosphere in 21st century. Sci. China Ser. D-Earth Sci. 51, 885–898. doi:10.1007/s11430-008-0039-y

Piao, S., Fang, J., Ciais, P., Peylin, P., Huang, Y., Sitch, S., Wang, T., 2009a. The carbon balance of terrestrial ecosystems in China. Nature 458, 1009–1013. doi:10.1038/nature07944

Shao, J., Zhou, X., Luo, Y., et al., 2016. Uncertainty analysis of terrestrial net primary productivity and net biome productivity in China during 1901–2005. J. Geophys. Res. Biogeosci. 121, 2015JG003062. doi:10.1002/2015JG003062

Tian, H., Melillo, J., Lu, C., Kicklighter, D., Liu, M., Ren, W., Xu, X., Chen, G., Zhang, C., Pan, S., Liu, J., Running, S., 2011a. China’s terrestrial carbon balance: Contributions from multiple global change factors. Global Biogeochem. Cycles 25, GB1007. doi:10.1029/2010GB003838

Zhu, X., Yu, G., He, H., et al., 2014. Geographical statistical assessments of carbon fluxes in terrestrial ecosystems of China: Results from upscaling network observations. Global and Planetary Change 118, 52–61. doi:10.1016/j.gloplacha.2014.04.003
